# Effect of Hempseed Cake (*Cannabis sativa* L.) Incorporation on the Physicochemical and Antioxidant Properties of Reconstructed Potato Chips

**DOI:** 10.3390/foods11020211

**Published:** 2022-01-13

**Authors:** Xiaoyu Feng, Guoxiao Sun, Zhongxiang Fang

**Affiliations:** School of Agriculture and Food, The University of Melbourne, Parkville, VIC 3010, Australia; xffe1@student.unimelb.edu.au (X.F.); guoxiaos@student.unimelb.edu.au (G.S.)

**Keywords:** hempseed cake, potato chips, antioxidant capacity

## Abstract

Hempseed (*Cannabis sativa* L.) cake is a by-product after cold-pressing of oil from the hempseed, which is rich in protein and fiber. This study investigated the effect of hempseed cake incorporation on the physicochemical and antioxidant properties of reconstructed potato chips. Varying levels of hempseed cake (0, 5%, 10%, 15%, and 20%) were added, and the results showed that the addition of hempseed cake at 20% level significantly increased the protein and total dietary fiber content from 2.74 ± 0.62 g/100 g to 9.66 ± 0.28 g/100 g and from 2.76 ± 0.31 g/100 g to 13.57 ± 0.42 g/100 g, respectively. In addition, a 20% reduction in lipid content was observed in the 20% hempseed cake addition group. Furthermore, lightness value (L*) was significantly reduced from 72.23 ± 1.22 to 46.40 ± 1.76, while the hardness was enhanced with a higher level of hempseed cake supplementation in the potato chips sample. Compared with the control (no hempseed cake), the supplement of 20% cake increased the total phenolic content from 0.19 ± 0.01 to 0.26 ± 0.01 mg GAE/g. The ABTS radical scavenging rate was also significantly enhanced with the increased levels of hemp cake. However, the peroxide value and TBARS results showed that the addition of hempseed cake accelerated the lipid oxidation in the sample, possibly due to the highly unsaturated fatty acid residues in the hempseed cake. The results suggested that more research is needed for the incorporation of hempseed cake in potato chips.

## 1. Introduction

Hempseed cake (*Cannabis sativa* L.) is one of the most underutilized oilseed cakes. Most people confuse the recreational drug of marijuana with industrial hemp, thereby the value and development of industrial hemp have not been extensively explored [1]. Depending on the variety, hempseeds typically contain 24–32% protein, 27–36% lipids, 32–37% carbohydrate, 29–37% fiber, and 5–6% ash [2,3,4]. Hempseeds can be considered as a rich source of protein since the protein content is comparable to soybean and higher than other similar products, such as flaxseeds (20.9%), quinoa seeds (20.9%), and buckwheat seeds (27.8%) [5]. In addition, hempseed protein is rich in edestin and essential amino acids and has excellent digestibility [6]. Hempseeds are also considered to be oilseeds rich in total polyphenols, whose total polyphenol content is slightly higher than that of linseed (5.88–10.63 mg CAE/g hempseed vs. 4.64–9.40 mg CAE/g of linseed) [7]. The two predominant phenolic compounds in hempseeds are xylolamide and hydroxycinnamic acid [8]. It is worth noting that in the whole hempseeds, polyphenols are mainly located in the hull instead of the caryopsis [9]. The utilization of hempseeds in the food industry is developing rapidly because of the growing awareness of the value of agri-food by-products and the negative impact of animal-derived protein on the environment [10]. The research on hempseeds in the food industry mainly includes the baking industry [11], extruded food [12], beverages [13], and meat products [14]. Hempseed cake is the by-product of the oil extraction process and is also rich in fiber, protein, polyphenols, vitamins, and essential amino acids [1]. Previous research has focused on adding hempseed cakes to corn snacks [15], pork patties [16], meatballs [17], bread [18], and crackers [19]. Results showed that the incorporation of hempseed cake usually increased the protein and dietary fiber content, influenced sensory properties such as the color, and altered the rheology features of bread [15,16,20]. However, hemp cake has not been widely used in the food industry and is usually disposed of as waste.

Potato chips (crisps) are one of the most popular snacks globally which are produced in two main types: one is traditional potato chips, which are made by directly slicing potatoes and deep-fried; the other is reconstituted potato chips (also referred as reconstructed potato chips or stackable potato chips), which are made of dough constituted with potato, starch, emulsifier, and water that is extruded or pressed into shape and then fried [21,22]. However, studies have found that snacks generally have the issues of high salt, high fat, high calories, and low nutritional value [23]. With the increasing awareness of a healthy lifestyle, consumers demand foods with higher nutritional value [24]. To produce healthier potato chips, current research is working on the incorporation of active substances or vegetable ingredients into potato snacks. For traditional potato chips, the method that can be adopted is vacuum impregnation. Tiwari et al. [25] used calcium chloride to fortify potato chips by vacuum impregnation under 15 mmHg vacuum pressure and obtained the products with 7.1 times higher calcium content than commercial products (700 mg/100 g), with acceptable organoleptic properties. In the case of reconstituted potato snacks, Lisiecka and Wójtowicz [26] added 30 g/100 g of fresh beetroot pulp to fried potato snacks, resulting in a final product with increased protein and soluble fiber content, as well as increased total phenolic content and antioxidant activity.

The aim of this research was to valorize of hempseed cake by incorporating different amounts (0–20%) into reconstructed potato chips; investigate the possibility of potato chips enrichment with hempseed cake; and determine the nutritional value, antioxidant capacity, and lipid oxidation rate of the product. The results showed that the addition of hempseed cake had positive effects on the nutritional and antioxidant values of the potato chips sample; however, it also accelerated the lipid oxidation of the product. This research could provide a valuable reference in the utilization of hempseed cake in new food product developments.

## 2. Materials and Methods

### 2.1. Materials

Hempseed cake powder was provided by an Australian commercial hemp producer (Australian Primary Hemp, Geelong, Australia). From the product label, it contains 49.8 g/100 g protein, 13.9 g/100 g lipid, 4.6 g/100 g carbohydrate, and 19.7 g/100 g dietary fiber. The powder was sealed in polyethylene bag under refrigerated condition (4 °C) before use. Potato starch, wheat flour, and salt were purchased from a local supermarket (Woolworths, Parkville, Australia). Other chemicals were purchased from Sigma-Aldrich (Castle Hill, Australia) and Chem-Supply (Parkville, Australia). Analytical-grade reagents were used in all experiments.

### 2.2. Sample Preparation

The potato chips were prepared based on the method of Miao et al. [27]. The recipe for a classic reconstructed potato chips dough (Table 1) was potato starch, wheat flour, and water, at a ratio of 3:1:2.5. The potato chips made with no hempseed cake addition were used as control. Other potato chips were made by replacing the mixture of potato starch and wheat flour with 5%, 10%, 15%, and 20% hempseed cake flour. Then, the powders were mixed to form the dough and compressed into 2 mm sheets by a dough-pressing machine (Kogan, Melbourne, Australia), then cut into thin slices of uniform shape (3.0 cm × 3.0 cm × 2.0 mm). The slices were placed in an oven at 50 °C for 30 min and then placed into an electric fryer (Contempo Deep Fryer 3L, Melbourne, Australia) with fresh canola oil and fried at 180 °C for 3 min. Then, the potato chips were placed on a wire screen to drain the oil for 5 min. After being cooled to room temperature, the potato chips were packed in a sealed plastic bag and stored at −20 °C before analysis.

### 2.3. Nutritional Value

The ash content is determined by the direct burning method [28]. The protein content determination was performed using the Kjeldahl method [29] and the lipid content using Soxhlet extraction method [30]. Soluble, insoluble, and total dietary fiber were analysed using a Total Dietary Fiber Assay Kit (Megazyme, Bray, Ireland) based on the AOAC method [31].

### 2.4. Physical Characteristics: Color and Texture

The method of color measurement of the potato chips was in accordance with Pojić, Dapčević Hadnađev, Hadnađev, Rakita, and Brlek [18]. A Minolta chromameter (Model CR-400; Konica Minolta Sensing Inc., Osaka, Japan) equipped with a standard light source D65 and an aperture size of 8 mm was used. Potato chips samples were ground into powder by a grinder (MultiGrinder™ II, Sunbeam, Australia), and the color was measured by closely attach to the chromameter. L* (lightness-darkness), a* (redness–greenness) and b* (yellowness–blueness) values were recorded. The instrument was calibrated with a standard white board (L* = 98.45, a* = −0.10, b* = −0.13) before use. A total of 9 readings were collected per sample.

The texture analysis (i.e., hardness) of the chips sample was evaluated at room temperature (∼20 °C) using a Lloyd LS5 universal testing machine (Ametek Inc., Berwyn, PA, USA) according to Segnini, et al. [32] whit minor modifications. Hardness, the maximum compression force, was measured by recording the peak force value of each sample at a puncture test. A three-point support of 15 mm distance was used to support the chips sample, and a 2 mm probe moving at a constant test speed of 60 mm/min was used to break the chips. The maximum force was obtained from the force vs. distance curves generated by the NEXYGENPlus software (Ametek Inc., Berwyn, PA, USA). A total of 9 chips were tested per treatment.

### 2.5. Total Phenolic Content (TPC)

TPC was measured using the Folin–Ciocalteu method described by Wang et al. [33], with some modifications. Briefly, 5 g sample was mixed with 25 mL methanol and shaken on a platform mixer (Ratek Instruments Pty Ltd., Boronia, Australia) for one hour. The mixture was centrifuged at 4000× *g* for 10 min at room temperature, and the supernatant was collected and filtered with a Whatman No. 1 filter paper. Filtrate of 0.5 mL and 2.5 mL Folin–Ciocalteu solution were mixed for 10 min, then 2 mL 7.5% sodium carbonate solution was added. The mixture was shaken for 30 min before the absorbance was measured at 760 nm by a UV-Vis spectrophotometer (Multiskan GO, Thermo Scientific, Vantaa, Finland). The standard curve was prepared using gallic acid with a concentration range of 0.01 to 0.1 mg/mL. The TPC in a sample was expressed as milligrams of gallic acid equivalent (GAE) per gram of sample. All tests were performed in triplicate.

### 2.6. Antioxidant Capacity–DPPH and ABTS Assay

The DPPH and ABTS radical scavenging antioxidant activities were measured according to the description of Leonard et al. [34]. For DPPH assay, 1 mL of the above filtrate and 4 mL of 0.1 mol DPPH solution were mixed and kept in the dark for 30 min. Methanol was used as a blank, and a mixture of methanol and DPPH was used as a control. The absorbance of the mixture was recorded at 515 nm using the UV-vis spectrophotometer. The percentage of DPPH inhibition rate was calculated using the following formula:

(1) $$\mathrm{DPPH}\;\mathrm{Inhibition}\;\left(\%\right)=\left(1-\frac{\mathrm{Abosorbance}\;\mathrm{of}\;\mathrm{sample}}{\mathrm{Abosorbance}\;\mathrm{of}\;\mathrm{control}}\;\right)\times 100\%$$

For ABTS assay, 10 mL of 7.4 mM ABTS solution and 10 mL of 2.6 mM potassium persulfate were mixed and kept in the dark for 12 h. The obtained stock ABTS solution was diluted with methanol to prepare an ABTS working solution with the absorbance of 1.1 ± 0.02. Then, 200 μL of the above filtrate was mixed with 1000 μL of ABTS working solution. After being shaken for 2 h in the dark, the absorbance of the mixture was measured at 734 nm using the UV-vis spectrophotometer. The percentage of ABTS inhibition rate was calculated using the following formula:

(2) $$\mathrm{ABTS}\;\mathrm{Inhibition}\;\left(\%\right)=\left(1-\frac{\mathrm{Abosorbance}\;\mathrm{of}\;\mathrm{sample}}{\mathrm{Abosorbance}\;\mathrm{of}\;\mathrm{control}}\;\right)\times 100\%$$

### 2.7. Lipid Oxidation–Peroxide Value and TBARS Assay

The peroxide value was measured according to the method of Okpala et al. [35], with slight modifications. Four-gram blended chips sample (M) was mixed with 10 mL chloroform and 15 mL glacial acetic acid, which were then filtered through a Whatman No. 1 filter paper. The filtrate was shaken vigorously for approximately 30 s, 1 mL of fresh saturated aqueous potassium iodide (KI) solution was added, and the sample was left in the dark for 5 min. Following this, 25 mL distilled water was added to release the iodine and then titrated with 0.01 M (T) of sodium thiosulphate solution (*V*_1_). The distilled water was used as blank. The PV was expressed in milli-equivalents of active oxygen per kilogram (mEq active O_2_/kg) of the sample, as determined using the following equation:

PV = (*V*_1_ − *V*_0_)*T* × 10^3^/M (3)

where the variable ‘*V*_1_’ represents the volume of sodium thiosulphate solution (mL), ‘*V*_0_’ represents the volume of the blank (mL), ‘M’ represents the mass of sample, and ‘*T*’ represents the molarity of sodium thiosulphate solution.

Lipid oxidation of potato chips was also evaluated by thiobarbituric acid reactive substances (TBARS) assay based on Xiong et al. [36], with slight modifications. Briefly, 5.0 g of sample was mixed with 10 mL of 10% trichloroacetic acid (TCA) solution and shaken in a homogeniser (Ultra-turrax T25 Digital Disperser, IKA Labortechnik, Staufen, Germany) for 1 min. The mixture was then filtered through a Whatman No. 1 filter paper, and 2 mL of filtrate was mixed with 2 mL of 0.02 M thiobarbituric acid (TBA), incubated at 95 °C for 30 min, and cooled with tap water. The absorbance at 532 nm was measured by the UV-vis spectrophotometer, and the non-specific turbidity at 600 nm was corrected. TCA solution was used as a blank, and 0–20 μM 1,1,3,3-tetraethoxypropane (TEP) was used as the standard. The results were expressed as milligrams of malondialdehyde per kilogram of chips sample (mg MDA/kg).

### 2.8. Data Analysis

The results were reported as mean ± SD. All data were analyzed by one-way ANOVA using Microsoft Excel (Microsoft Corporation, Redmond, WA, USA) with the hempseed cake addition as a factor. Differences between means were calculated using Fisher LSD test at a *p* < 0.05 significance level.

## 3. Results and Discussion

### 3.1. Nutritional Value

After incorporation with hempseed cake, the ash, protein, and dietary fiber content in potato chips increased while the lipid content decreased (Table 2). The substitution of hempseed cake had a significant effect (*p* < 0.05) on the protein content of the potato chips, which increased from 2.74 ± 0.62 g/100 g in the control to 9.66 ± 0.28 g/100 g in the sample when 20% of hempseed cake was added (H20). Enriching food with protein has been one of the main product development trends of the food industry due to the inadequate dietary protein intake of some people in under-developed countries/areas, which is a good approach to prevent protein malnutrition [37]. The increase of the protein content in the hempseed-cake-added samples is due to the high protein content of the hempseed cake in this study (49.8%, in Section 2.1.) and another report [38]. This is similar to the observations of Norajit, Gu, and Ryu [12], who studied an energy bar made from an extruded rice hemp mixture, to which 20% defatted hemp flour was added to boost the protein content from 6.16 ± 0.08 to 10.10 ± 0.03 g/100 g.

Table 2 also shows that compared with the control, the replacement of 20% hempseed cake significantly reduced the amount of lipids from 30.83 ± 4.52 g/100 g to 24.53 ± 1.03 g/100 g and significantly increased the total dietary fiber content from 2.76 ± 0.31 g/100 g to 13.57 ± 0.42 g/100 g (*p* < 0.05). In addition, the results showed that the content of insoluble dietary fiber in the crisp samples was higher than that of soluble fiber, due to the fact that the dietary fiber in hempseeds consists mainly of insoluble dietary fiber [39]. The rise in dietary fiber content is noteworthy due to the beneficial effects of dietary fiber on reducing appetite, improving insulin sensitivity, and reducing low-density lipoprotein (LDL) and cholesterol levels [40].

Conventional potato chips typically have a lipid content of 30%, and high lipid content is associated with obesity and coronary heart disease, thus posing a health risk [41]. The characteristics of potato chips (thickness, density, surface roughness, and material composition), pretreatment, and processing conditions are related to oil absorption of potato chips [42]. In this study, the incorporation of low-level hempseed cakes slightly increased the lipid from 30.83 ± 4.52 g/100 g of control to 31.15 ± 1.35 g/100 g of H10. The possible explanation is that the hempseed cake contained a certain amount of lipids (13.9 g/100 g). However, the control sample (no hempseed addition) had a lipid content of 30.83 ± 4.52 g/100 g, which indicated that the fat content in the potato chip sample was mainly derived from the absorption of frying oil rather than the addition of raw materials. Furthermore, as the amount of hempseed further increased, the lipid content of the sample decreased, and the H20 result was significantly lower than others (Table 2). The reduction of lipid content may be attributed to the interaction between the protein and dietary fibers provided by the hempseed cake and the starch in the mixture to form a tighter protein-carbohydrate matrix, thereby reducing the infiltration of oil during the frying process [19]. Yang, et al. [43] also found that the increase in protein content affected the surface and internal structure of the recombinant potato chips and that the protein concentration is inversely proportional to the fat content. Furthermore, the addition of fresh leek pulp significantly reduced the lipid content of fried reconstituted potato snacks, which the authors attributed to the lesser oil absorption caused by lower carbohydrate content and higher fiber and protein content of fresh leeks [44]. However, in gluten-free biscuits, 20–60% of corn flour was replaced by hemp flour, and the results showed that 20% hemp flour replacement had no significant effect on the fat content of the biscuit samples (*p* > 0.05) [45]. The discrepancy may be due to the lower protein content of the hemp powder in this research (19.7 g/100 g) [45] compared to the present experiment (49.8 g/100 g).

### 3.2. Physical Characteristics

**Figure 1:**
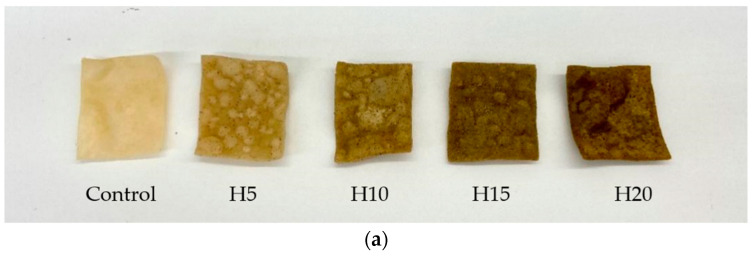

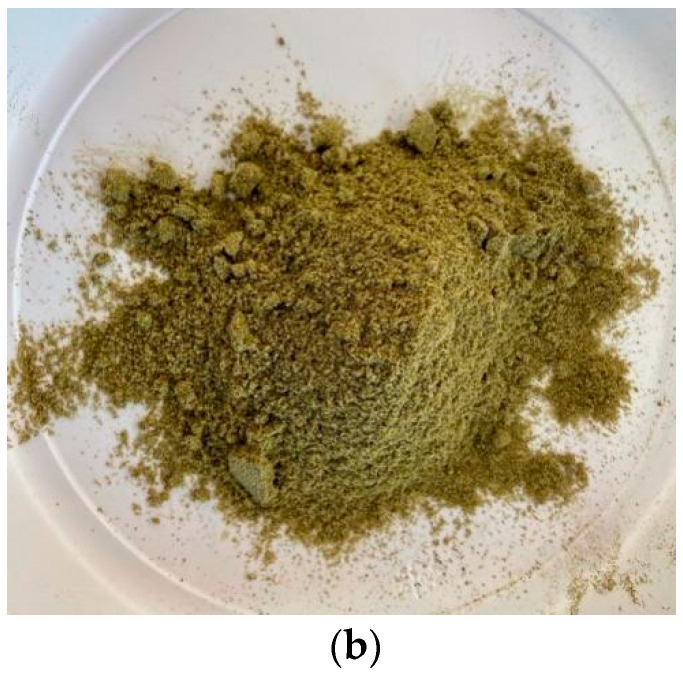
(**a**) Photograph of potato chips with different levels of hempseeds cake. (**b**) Photograph of hempseeds cake flour used. Control = potato chips made by wheat flour and potato starch only; H5 = 5% hempseed cake replacement; H10 = 10% hempseed cake replacement; H15 = 15% hempseed cake replacement; and H20 = 20% hempseed cake flour replacement.

Color is considered an important food qualitative indicator since it plays a key role in consumers’ perception and acceptability [46]. According to the results, the lightness (L*) of potato chips decreased significantly with a higher level of hemp cake supplementation (Table 3 and Figure 1a), mainly because of the darker color of hempseed cake powder (Figure 1b). This result is consistent with a previous study where Kotecka-Majchrzak, Kasałka-Czarna, Spychaj, Mikołajczak, and Montowska [17] added different amounts of hempseed cake (0.9%, 2.6%, 4.2%, and 7.4% *w*/*w*) to the meatballs and observed a significant decrease in lightness. Similarly, Norajit, Gu, and Ryu [12] reported that the extruded rice flour mixture with the addition of hemp flour was darker compared to the control, with the lowest L* showing at 40% level of whole hemp flour addition.

For the redness (a*)*,* except for sample H10, the a* values of H5, H15, and H20 were significantly enhanced compared with the control (*p* < 0.05). This was not expected since the hemp cake flour used in the experiment was green in color (Figure 1b), and it was predicted that the redness a* value should decrease with increasing amounts of hemp cake. A possible explanation is that at hempseed cake additions of 10%, the green color of the cake (Figure 1b) was complexed with the potato dough, which was reflected by a relatively lower a * value than other hempseed cake added samples but still higher than the control (Table 3). The enhancement of red color of all hempseed-cake-incorporated samples could be due to the increased protein content, which may have reacted with reduced sugars (Maillard reaction) to produce brown pigments during high-temperature processes such as frying [47]. This can also be seen in the upward trend of the b* value, which represents the blueness-yellowness. In addition, Wang and Xiong [48] reported that the hemp protein isolate was dark green to brown when exposed to molecular oxygen, which may also be one of the reasons for the color change of the potato chips sample.

The texture is another important factor in assessing food quality. In the case of potato chips, crispness is highly correlated with consumer satisfaction [49]. As shown in Table 3, the addition of hempseed cake to the chips significantly increased the hardness of the chips (*p* < 0.05). This could also be explained by the previously mentioned formation of a denser network by proteins and carbohydrates [19]. In a previous study, Sharma and Prabhasankar [20] also observed that the addition of 10% hempseed cake increased the hardness of the pasta from 2.93 N to 5.8 N. The author explained that this phenomenon was due to the increased protein content in the product.

### 3.3. Antioxidant Capacity

The results of the antioxidant properties are summarized in Table 4. Firstly, incorporation of hempseed cake significantly increased the TPC in potato chips, from 0.19 ± 0.01 mg GAE/g of control to 0.26 ± 0.01 mg GAE/g of H20. Hempseeds are rich in phenolic compounds, and most of the polyphenols are present in the hull that is left over in hempseed cake after oil extraction [34]. Furthermore, the increase in antioxidant activity may also be due to the antioxidant capacity of melanoid pigments such as hydroxymethylfurfural, produced by the Maillard reaction during the thermal process [50]. Similar results were reflected in a previous study when 20% hemp flour was added to the cookie sample, and the TPC was increased significantly from about 0.41 to 1.22 mg GAE/g [51]. Similarly, the addition of 15% hempseed flour significantly increased the TPC value of wheat bread from 256.43 to 563.63 mg GAE/kg sample [11].

In terms of DPPH and ABTS free radical assays (Table 4), results showed that the influence on DPPH inhibition activity was not significant, while there was a significant increase in ABTS inhibition rate (*p* < 0.05). These results are inconsistent with the previous studies in which the DPPH free radical scavenging activity of extruded rice/hemp energy bars was increased significantly with the addition of defatted hemp flour (20%, 30%, and 40%) [12]. The possible reason for the discrepancy is that the level of hempseeds is different, with the addition of 20–40% defatted hemp flour in other studies compared with the addition of 5–20% hempseed cake in this one. In terms of the differences in the antioxidant capacity of potato chips using the DPPH and ABTS assays, a previous study found that the antioxidant capacity determined by ABTS assay is significantly higher than that of DPPH, and ABTS assay could be better reflect the antioxidant capacity of various foods [52].

### 3.4. Lipid Oxidation

Both the PV assay and TBARS assay were used to estimate the degree of oxidation of lipids in the products. As shown in Table 5, the ranges of PV and TBARS values are 0.75–6.80 meq O_2_/kg and 0.27–0.78 mg MDA/kg, respectively. According to previous studies, the PV and TBARS values of fresh potato chips were in the vicinity of 1.69 meq O_2_/kg and 1.3 ± 0.5 mg MDA/kg [53,54]. The main reason for the quality deterioration of potato chips is the oxidation of lipids absorbed during frying [55]. Generally, the PV of fresh vegetable oil is less than 10 meq O_2_/kg oil [56] and the food with TBA higher than 1–2 μmol MDA/g oil has a rancid smell [57]. As shown in Table 5, the PV value and TBARS value of all potato chips samples are below the limit, suggesting these products are acceptable in terms of lipid quality. In addition, the trend for PV and TBARS change was generally consistent, with both showing an increase. For peroxide value, the addition of 5% and 10% hempseed cake did not show a significant difference from the control, while that in H15 and H20 was significantly higher but not between these two groups. In contrast, the TBARS value was significantly higher after 5% hempseed cake addition (*p* < 0.05) and even higher with more hempseed cake supplementation (Table 5).

The increase in PV and TBARS values indicate that the addition of hempseed cake accelerated the lipid oxidation in potato chips. This result is consistent with a previous study in which the addition of 1.5–2.0% hempseed cake increased the TBARS value and oxidation rate of pork patties [16]. In addition, Xiong, et al. [58] added polyphenol-rich sorghum bran to beef sausages and observed the promoted lipid oxidation. One possible reason could be that the hempseed oil is composed of 90% easily oxidisable polyunsaturated fatty acids [8]. During the chip-frying process, the oxidation of polyunsaturated fatty acids could have accelerated the lipid oxidation process, although the lipid content of the hempseed cake was only 11% [38]. Another explanation could be that the phenolic compounds in the hempseed cake might have a pro-oxidant activity under the 180 °C frying condition in the presence of redox metals, which may come from the electric fryer [59]. In order to inhibit lipid oxidation in potato chips, future research could focus on adding natural antioxidants to the product. For example, Kerner, Jõudu, Tänavots, and Venskutonis [16] added 0.5% sweetgrass ethanol extract as natural antioxidant to pork patties incorporated with raw hempseed, and they observed a positive effect on inhibiting lipid oxidation.

## 4. Conclusions

The addition of 20% hempseed cake to reconstituted potato chips significantly increased the protein and total dietary fiber levels from 2.74 ± 0.62 g/100 g to 9.66 ± 0.28 g/100 g and from 2.76 ± 0.31 g/100 g to 13.57 ± 0.42 g/100 g, respectively, and significantly reduced the fat content from 30.83 ± 4.52 g/100 g to 24.53 ± 1.03 g/100 g, due to reduced absorption of frying oil. The lightness value (L*) was significantly reduced, while the hardness was enhanced with a higher level of hempseed cake supplementation in the potato chips sample. Furthermore, the results of TPC and ABTS show that the addition of hempseed cake significantly increases the polyphenol content in the reconstituted potato chips and enhances their antioxidant properties. However, according to the PV and TBARS values, the incorporation of hempseed cake accelerated the lipid oxidation in the potato chips, possibly due to the oxidation of highly unsaturated fatty acids in the hempseed cake during high-temperature frying. Future research could investigate the possibility of incorporating natural antioxidants to the reconstructed potato chips to inhibit lipid oxidation; the methods of reducing the oil residual in the hempseed cake to improve its resilience under harsh food processing conditions (e.g., high temperature, oxygen exposure); and the effect of the integration of hempseed cake on the content of acrylamide in fried snacks since acrylamide is one of the most common carcinogens in fried foods. In addition, a sensory analysis should be carried out to assess consumers’ acceptability of the new food products.

## Figures and Tables

**Table 1:** Recipe of hempseed cake incorporated potato chips.

Group	Hempseed Cake (g)	Potato Starch (g)	Wheat Flour (g)	Water (mL)	Salt (g)
Control	0	60	20	50	2
H5	4	57	19	50	2
H10	8	54	18	50	2
H15	12	51	17	50	2
H20	16	48	16	50	2

H5 = 5% hempseed cake addition, H10 = 10% hempseed cake addition, H15 = 15% hempseed cake addition, and H20 = 20% hempseed cake addition.

**Table 2:** Nutritional characteristics of potato chips with incorporation of hempseed cake (mean ± SD, *n* = 3).

	Control	H5	H10	H15	H20
Ash (g/100 g)	2.22 ± 0.01 ^d^	2.91 ± 0.17 ^c^	3.44 ± 0.04 ^bc^	3.86 ± 0.14 ^ab^	4.41 ± 0.22 ^a^
Protein (g/100 g)	2.74 ± 0.62 ^d^	3.52 ± 0.39 ^d^	5.72 ± 0.61 ^c^	7.50 ± 0.43 ^b^	9.66 ± 0.28 ^a^
Lipid (g/100 g)	30.83 ± 4.52 ^ab^	30.10 ± 2.05 ^ab^	33.94 ± 5.21 ^a^	31.15 ± 1.35 ^ab^	24.53 ± 1.03 ^b^
Soluble fiber (g/100 g)	1.20 ± 0.31 ^d^	2.15 ± 0.51 ^c^	2.33 ± 0.19 ^bc^	2.97 ± 0.06 ^b^	4.01 ± 0.19 ^a^
Insoluble fiber (g/100 g)	1.56 ± 0.01 ^e^	2.68 ± 0.44 ^d^	4.52 ± 0.24 ^c^	6.01 ± 0.62 ^b^	9.56 ± 0.23 ^a^
Total Dietary fiber (g/100 g)	2.76 ± 0.31 ^e^	4.83 ± 0.95 ^d^	6.85 ± 0.43 ^c^	8.98 ± 0.68 ^b^	13.57 ± 0.42 ^a^

Control = potato chips made by wheat flour and potato starch only; H5 = 5% hempseed cake addition; H10 = 10% hempseed cake addition; H15 = 15% hempseed cake addition; and H20 = 20% hempseed cake addition. Values in the row with different superscript letters are significantly different at *p* < 0.05.

**Table 3:** The color and texture properties of potato chips with incorporation of hempseed cake (mean ± SD, *n* = 9).

Variations	Control	H5	H10	H15	H20
L*	72.23 ± 1.22 ^a^	64.00 ± 1.81 ^b^	56..48 ± 1.65 ^c^	47.86 ± 1.01 ^d^	46.40 ± 1.76 ^e^
a*	0.20 ± 0.07 ^c^	0.99 ± 0.16 ^a^	0.77 ± 0.20 ^b^	1.15 ± 0.12 ^a^	1.03 ± 0.18 ^a^
b*	21.23 ± 0.91 ^d^	23.71 ± 1.15 ^c^	24..91 ± 1.32 ^bc^	24.17 ± 0.88 ^bc^	25.32 ± 0.90 ^a^
Hardness (N)	5.38 ± 3.08 ^c^	12.51 ± 6.20 ^b^	12.96 ± 3.84 ^b^	19.41 ± 4.59 ^a^	20.06 ± 6.06 ^a^

Control = potato chips made by wheat flour and potato starch only; H5 = 5% hempseed cake replacement; H10 = 10% hempseed cake replacement; H15 = 15% hempseed cake flour replacement; and H20 = 20% hempseed cake flour replacement. The values of different superscript letters in the same row are significantly different (*p* < 0.05).

**Table 4:** The antioxidant capacity of potato chips with incorporation of hempseed cake (mean ± SD, *n* = 3).

	Control	H5	H10	H15	H20
TPC (mg GAE/g)	0.19 ± 0.01 ^d^	0.21 ± 0.002 ^cd^	0.23 ± 0.005 ^bc^	0.25 ± 0.01 ^ab^	0.26 ± 0.01 ^a^
DPPH inhibition rate (%)	18.76 ± 2.87 ^a^	19.62 ± 1.29 ^a^	23.52 ± 3.22 ^a^	24.06 ± 1.50 ^a^	23.22 ± 1.61 ^a^
ABTS inhibition rate (%)	27.53 ± 1.71 ^d^	31.87 ± 1.46 ^c^	42.98 ± 3.03 ^b^	46.37 ± 2.94 ^ab^	47.76 ± 2.23 ^a^

Control = potato chips made by wheat flour and potato starch; H5 = 5% hempseed cake replacement; H10 = 10% hempseed cake flour replacement; H15 = 15% hempseed cake replacement; and H20 = 20% hempseed cake replacement. Values in the same row with different superscript letters are significantly different at *p* < 0.05.

**Table 5:** The lipid oxidation of potato chips with incorporation of hempseed cake (mean ± SD, *n* = 3).

	Control	H5	H10	H15	H20
Peroxide value (meq O_2_/kg)	0.75 ± 0.36 ^b^	2.05 ± 0.67 ^b^	2.30 ± 0.15 ^b^	5.40 ± 0.11 ^a^	6.80 ± 0.62 ^a^
TBARS (mg MDA/kg)	0.27 ± 0.01 ^d^	0.47 ± 0.06 ^c^	0.49 ± 0.04 ^bc^	0.58 ± 0.03 ^b^	0.78 ± 0.04 ^a^

Control = potato chips made by wheat flour and potato starch; H5 = 5% hempseed cake replacement; H10 = 10% hempseed cake flour replacement; H15 = 15% hempseed cake replacement; and H20 = 20% hempseed cake replacement. Values in the same row with different superscript letters are significantly different at *p* < 0.05.

## Data Availability

The data presented in this study are available in article.
